# Establishing the relationship between history of childhood trauma and personality disorder using the ICD‐11 classification system

**DOI:** 10.1002/pmh.1648

**Published:** 2024-11-28

**Authors:** Jordan Lock, Min Yang, Peter Tyrer

**Affiliations:** ^1^ University of Lincoln Lincoln UK; ^2^ Faculty of Health, Art and Design Swinburne University of Technology Melbourne Australia; ^3^ West China School of Public Health Sichuan University West China Medical Centre, Sichuan University Chengdu China; ^4^ Division of Psychiatry Imperial College London UK; ^5^ Faculty of Social Sciences Nottingham Trent University Nottingham UK

## Abstract

The temporal relationship between childhood trauma and personality status using the ICD‐11 classification was examined in 75 adults (65%F) aged between 19 and 72 selected by constrained procedure to ensure the full range of personality disturbance. Trauma history was assessed using the Childhood Traumatic Events Scale, past and recent versions and personality status using scales to assess the severity and domains of the ICD‐11 system, PDS‐ICD‐11 and PAQ‐11. The PDS‐ICD‐11 findings showed 52 (59.3%) had some personality disturbance with 17 (22.7%) having moderate or severe personality disorder. There was a significant association between childhood sexual trauma and ICD‐11 personality severity (*p* = 0.005) and a lessened association with recent trauma (*p* = 0.02). Apart from the borderline composite score, the PAQ‐11 negative affectivity domain was significantly associated with childhood sexual trauma (*p* = 0.003), but other domains were not linked. The Anankastia domain was significantly associated with early major physical illness (*p* = 0.012), and physical illness both in early life (*p* = 0.005) and recent exposure (*p* = 0.024) were associated with personality disorder. Other forms of childhood abuse and illness or injury in childhood did not differ with regard to later personality disorder. The results confirm a significant but not overwhelming relationship between childhood sexual trauma and severity of ICD‐11 personality disturbance in adult life that is greater than for other classes of trauma.

## INTRODUCTION

Childhood adversity is very commonly associated with mental disorder. Kessler et al. (2010) examined world data and found that nearly 30% of mental disorders were preceded by major problems in childhood, usually associated with parental mental illness, child abuse and neglect. Trauma is a significant component of this adversity and repeated episodes of childhood trauma are associated with a greater incidence of mental disorder (McKay et al., 2021). Personality disorder in general has also been associated with childhood trauma with most studies finding that antisocial and borderline characteristics have the strongest associations (Berenz et al., 2013; Dargis et al., 2016). This view has been further developed into an aetiological link; specifically, that borderline personality disorder is a direct consequence of childhood abuse. In an early study, Herman et al. (1989) found that significantly more subjects with borderline personality disorder (81%) gave histories of such trauma, including physical abuse (71%), sexual abuse (68%) and witnessing serious domestic violence (62%) than those with no personality disorder or with borderline traits only. Herman (1992) subsequently made the case that this association was so common that it was inappropriate to continue to diagnosis such early trauma sufferers as personality disordered and more appropriate to give them a new diagnosis, complex post‐traumatic stress disorder, and this has now been adopted in the ICD‐11 classification (Maercker et al., 2022). Although much further work has amplified the links between abuse and personality disorder (Bozzatello et al., 2021; van der Kolk et al., 1994), almost all studies have concentrated on the borderline group only.

Childhood sexual abuse and other personality disorders have also been studied closely. But the results have been mixed. A recent review found a large range of 16% to 86% incidence of personality disorder after sexual abuse (De Aquino Ferreira et al., 2018) and Fossati et al. (1999) found a modest association only that was not sufficient to link cause and effect. In the Collaborative Longitudinal Personality Disorders Study, a higher incidence of sexual abuse was found in patients with borderline personality disorder than in other personality disorders, although only paranoid, schizotypal and self‐defeating personality disorders were included in this comparison (Yen et al., 2002).

In this setting of uncertainty, it was therefore considered to be an appropriate time to compare the incidence of all forms of childhood trauma in a population covering the range of personality pathology. The ICD‐11 classification of personality disorder does not include any of the categorical diagnoses from DSM or previous ICD classifications. Although a borderline specifier was introduced as an optional extra (Tyrer et al., 2019), it is not a diagnosis. It was felt to be of value to determine the association between childhood trauma, including sexual abuse, and both the severity of ICD‐11 personality disorders and the association of trauma with each of the five domains that qualify the severity of disorder. This allows all shades of personality dysfunction to be examined in relationship to childhood trauma.

## METHODS

Because the ICD‐11 classification has had limited studies of its value the study addressed the hypothesis that childhood trauma and sexual abuse is a precursor of adult personality disorder and that borderline symptomatology was specifically associated with trauma. If there was a clear association the hypothesis would also predict that more severe trauma would be associated with more severe personality pathology. We therefore assessed both the diagnosis of personality disorder on the severity scale between personality difficulty and severe personality disorder and each of the five domains of personality, including an additional combination of borderline pathology found by Kim et al. (2021).

### Participants

In order to obtain a representative sample of all levels of severity of personality disorder, a constrained sample of participants was chosen from those currently receiving care for mental illness and/or personality disorder by PT and also those known to JL, selectively recruited by PT and JL to cover the extended range of personality dysfunction from normal personality to severe disorder.

### Assessments

The assessments of personality status in the study were relatively new and developed with the ICD‐11 classification in mind. The measurement of trauma questionnaires used was the Childhood Traumatic Events Scale (CTES) and the Recent Traumatic Events Scale (RTES). The CTES is a well‐established scale for assessing childhood traumatic events that occurred prior to the age of 17 (Pennebaker & Susman, 1988), with all aspects of trauma covered, including death of a family member or very close friend, parental divorce or separation, traumatic sexual experience, exposure to violence, extreme illness or injury and any other major upheaval. The RTES is similar to the CTES but with a different time frame covering the previous 3 years. The domains are generally the same, but parental separation has been removed and changed for separation/divorce from a spouse or significant other and there is a new category of job change.

Personality assessment was recorded with two measures linked to ICD‐11, the Personality Disorder Severity Scale (PDS‐ICD‐11) (Bach et al., 2021) and the Personality Assessment Questionnaire (PAQ‐11) (Kim et al., 2021).

### Personality Disorder Severity ICD‐11 (PDS‐ICD‐11) Scale

This short self‐report measure was developed in line with the official ICD‐11 classification (Bach et al., 2021). The questionnaire consists of 14 questions with a score of 17 suggesting significant personality dysfunction (Bach et al., 2021) with revised cut‐off thresholds for each of the different levels of personality dysfunction recently derived in a Danish population study with scores of 12, 16 and 19 for each level of severity (Bach et al., 2023). It has close correspondence with the DSM‐5 LPFS (Levels of Personality Function Scale) in the Alternative Model of Personality Disorder (AMPD) (Weekers et al., 2023).

### Quick personality assessment schedule for the ICD‐11 (PAQ‐11)

The PAQ‐11 was developed to be used as a tool to assess the five trait domains identified in ICD‐11 (Kim et al., 2021). The questionnaire consists of 17 questions each scored between 0 and 4. The PAQ‐11 is not used to assess severity, but high scores are linked to severity. Each question is related to the five different ICD‐11 trait domains; negative affectivity, Anankastia, dissociality, disinhibition and detachment. The PAQ‐11 was developed using some items derived from the Personality Assessment Schedule ICD‐11 version (PAS‐ICD‐11) (Kim et al., 2021) and it has good construct validity (Sellbom et al., 2023).

The PAQ‐11 assesses the five domains of personality disorder but is not a diagnostic tool as the domains are only qualifiers in ICD‐11. But they are likely to correlate highly with personality dysfunction when scores are raised. Although borderline personality disorder is not a diagnosis in ICD‐11, the PAQ‐11 has a borderline composite score covering this pathology (Kim et al., 2021) was included.

#### Statistics

Descriptive statistics were used to examine differences in individual characteristics such as age, sex and previous psychiatric diagnosis of participants by level of personality dysfunction (PD).

To examine the association between trauma and PD level, we firstly used the F‐test to compare mean difference of total trauma scores among levels of PD with ETA coefficient (define) to determine linearity in the mean score by personality level. Subsequently, a multinomial regression model for ordered data was used to test the impacts of trauma scores on the ordered personality level. Age, sex and previous diagnosis were again taken into account in the model analysis.

To test how each subtype of trauma could have impact on PD, taking into account the intercorrelation among the trauma types, we used multivariate logistic regression analysis a in multilevel modelling framework (Yang et al., 2000). This analysis estimates association between the PD measures (dimensional score or status) and each type of trauma simultaneously with a couple of important advantages. Firstly, by modelling multiple trauma items (six for the CTES and seven for the RTES) simultaneously, this analysis increases data points in the model and hence improved the statistical power for the estimation on association. For example, for the CTES types, there are six items *75 individuals = 450 data points effectively used to test the association, instead of 75 data points. Second, the multiple models take into account intercorrelation among the trauma subtypes in a variance–covariance structure, which will be used in the generalized Ward tests for the significance of the association between each trauma subtype and the PD condition. Hence, no other adjustment is needed for the multiple tests among the trauma types. This analysis was performed for the CTES and RTES items separately on each of the total scores of PDS‐ICD‐11, of PAQ‐11 borderline, and each of the five domain personality status, respectively. Age, sex and previous diagnose of individuals were adjusted in the analysis.

All tests were two‐sided with significance level at 0.05. The package IBM SPSS Statistics Version 26 was used for descriptive analyses and the package MLwiN 3.02 was used for modelling analysis in the study.

In this study, specifically linked to the ICD‐11 classification system, we wanted to test the following general hypotheses:

- Are there types of childhood and recent trauma specifically associated with adult personality dysfunction?
- Is trauma associated more strongly associated with more severe personality dysfunction?
- Which domains of personality are linked to childhood and recent trauma?

## RESULTS

### Participants

Seventy‐five subjects were recruited to the study. These were selected to cover the full spectrum of personality in ICD‐11 and included patients seen by a mental health charity, NIDUS‐UK, involved in nidotherapy (Tyrer et al., 2003), others who had episodes of mental illness in the past and others from the community to ensure a wide age range (Table 1). Because only a minority of the population has moderate or severe personality disorder, a constrained selection system was used to reduce skewing.

**TABLE 1:** Distribution of subjects by personality status in study.

ICD‐11 severity level	Number (%)	Gender	Mean age (SD)	No. previous mental illness (%)
No personality dysfunction	23 (30.7)	13 M/10F	40.2 (19.2)	7 (33.3)
Personality difficulty	14 (18.7)	2 M/12F	25.9 (18.4)	4 (30.8)
Mild personality disorder	21 (28.0)	8 M/15F	24.9 (12.3)	10 (47.6)
Moderate personality disorder	9 (12.0)	2 M/7F	24.4 (13.4)	6 (66.7)
Severe personality disorder	8 (10.7)	0 M/8F	26.9 (11.5)	6 (85.7)
Totals	75 (100.0)	23 M/52F	30.0 (16.8)	33 (46.5)

### Assessment of severity of personality disorder

We used the cut‐off points found in the recent Danish population study on ICD‐11 severity levels with a score of 12 indicating mild personality disorder, 16 moderate disorder and 19 severe disorder (Bach et al., 2023). We also used a Delphi study with 12 of the subjects on which we had personality status assessed by clinical interview to identify a score of 8 as personality difficulty.

Although the domain scores are not pathognomonic for personality disorder they are clearly related. A strong correlation was found between the total PAQ‐11 and PDS‐ICD‐11 scores (0.72). The borderline component of the PAQ‐11 was correlated significantly with the negative affectivity domain (0.88), detachment (0.66), disinhibition (0.32) and Anankastic domains (0.42).

Trauma scores, in particular when using the CTES scale, increased as the severity level of personality disorder increased. The linearity or correlation measure of trauma scores by PD severity level was moderate between 0.346 and 0.405 (Table 2).

**TABLE 2:** Mean scores on the Childhood Trauma Events Scale (CTES) and Recent Trauma Events Scale (RTES) in levels of personality dysfunction recorded using the Personality Disorder Severity ICD‐11 Scale (PDS‐ICD‐11).

PD severity level	*N*(%)	CTES mean (SD)	RTES mean (SD)
None	23 (30.7)	1.17 (1.15)	1.17 (0.89)
Difficulty	14 (18.7)	1.57 (1.02)	1.79 (1.31)
Mild	21 (28.0)	1.67 (1.39)	1.57 (1.12)
Moderate	9 (12.0)	2.22 (1.20)	2.11 (1.69)
Severe	8 (10.7)	3.00 (1.77)	2.50 (1.19)
*p*‐Value		0.013	0.06
Linearity (ETA)		0.405	0.346

*Note*: The Eta coefficient is a correlation measure between a continuous variable (such as the trauma score) and a discrete variable (such as level of personality).

Abbreviation: PD, personality dysfunction.

There were modest but significant correlations between total CTES scores and scores on the PDS‐ICD‐11 scale and for the negative affective, detachment and borderline scores on the PAQ‐11 domains. Less agreement was found for recent traumatic events and domain scores, but the PDS‐ICD‐11 correlation retained significance (Table 3).

**TABLE 3:** Pearson's correlation coefficients between personality domains (negative affectivity, detachment, Anankastia, disinhibition, dissociality and borderline composite measure (Bdl) and trauma scores on the Childhood Trauma Events Scale (CTES) and Recent Trauma Events Scale (RTES) (*N* = 75)).

		PAQ total	Neg Aff	Detac	Anank	Disinh	Dissoc	Bdl	PDS total
CTES	Pearson's	0.207	0.259	0.242	0.007	−0.008	−0.071	0.247	0.386
	Sig.(2‐sided)	0.075	0.025	0.037	0.950	0.949	0.547	0.033	0.001
RTES	Person's	0.263	0.221	0.276	0.074	0.115	0.020	0.221	0.307
	Sig.(2‐sided)	0.023	0.057	0.017	0.526	0.324	0.865	0.056	0.007

*Note*: PAQ & PDS total are the summed scores on the PAQ‐11 and PDS‐ICD‐11 scales.

### Sub‐types of trauma and personality status

The relationship between the individual types of trauma and ICD‐11 severity scores as recorded using the PDS‐11 scale, and with the borderline composite score (PAQ‐BD) showed a markedly significant association with early sexual.

Abuse but this was attenuated markedly after adjustment for age, sex and previous mental state diagnosis (Table 4). Early major illness remained a significant association. Further examination of the relationship between subtypes of trauma and domain scores in the ICD‐11 classification showed that negative affectivity was significantly associated with early sexual abuse and major illness before 17 years old and that the Anankastia domain was linked to major illness in childhood (Table 5). Some other associations did not reach significance, possibly because the numbers were small. The domains of Anankastia, disinhibition and dissociality had much lower associations than negative affectivity.

**TABLE 4:** Association between PDS‐ICD‐11, PAQ‐BD dimensional scores and the absence of subtype trauma with adjustment for age, sex and previous diagnosis.

**Table:** PDS‐ICD‐11 PAQ BD Est. (SE) *p*‐Value Est. (SE) *p*‐Value CTES Bereavement before 17 years old 0.051 (0.044) 0.247 0.036 (0.060) 0.561 Parental upheaval before 17 years old 0.067 (0.044) 0.129 0.087 (0.060) 0.149 Sexual violence before 17 years old 0.163 (0.057) 0.004 0.247 (0.088) 0.005 Nonsexual violence before 17 years old 0.075 (0.059) 0.206 0.133 (0.089) 0.135 Major illness before 17 years old 0.196 (0.071) 0.005 0.278 (0.123) 0.024 Other major upheaval before 17 years old 0.025 (0.043) 0.563 −0.004 (0.058) 0.944 RTES Bereavement past 3 years 0.003 (0.043) 0.952 0.019 (0.058) 0.748 Major upheaval in relationship past 3 years 0.038 (0.065) 0.555 −0.012 (0.089) 0.890 Traumatic sexual violence past 3 years 0.173 (0.075) 0.021 0.044 (0.090) 0.624 Nonsexual violence last 3 years 0.130 (0.077) 0.092 0.151 (0.105) 0.152 Extremely ill or injury last 3 years 0.028 (0.055) 0.605 0.032 (0.077) 0.682 Major change in work last 3 years 0.087 (0.047) 0.061 0.121 (0.063) 0.056 Other major upheaval last 3 years −0.011 (0.052) 0.831 −0.025 (0.070) 0.719

*Note*: The Est. in the table stands for the estimated difference in the mean scores of personality dysfunction measures in the logit scale between the presence and absence of the trauma condition. The greater the positive value the greater association between that particular trauma and personality disturbance.

Abbreviation: CTES, Childhood Traumatic Events Scale.

**TABLE 5:** Association between ICD‐11 domain status and previous life experiences with adjustment for age, sex and previous diagnosis.

Trauma subtype	Detachment	Negative affectivity	Anankastia	Disinhibition	Dissociality
	Est. (SE): *p*‐value	Est. (SE): *p*‐value	Est. (SE): *p*‐value	Est. (SE): *p*‐value	Est. (SE): *p*‐value
CTES					
Bereavement before 17 years old	0.156(0.609): 0.797	0.375(0.547): 0.493	0.170(0.518): 0.743	−0.251(0.540): 0.643	−0.853(0.732): 0.244
Parental upheaval before 17 years old	0.396(0.590): 0.502	0.360(0.546): 0.509	0.273(0.505): 0.588	0.320(0.520): 0.539	−.627(0.692): 0.365
Sexual abuse before 17 years old	−0.221(0.716): 0.758	1.191(0.641): 0.003**	0.709(0.584): 0.225	−0.199(0.607): 0.743	1.495(1.053): 0.156
Nonsexual abuse before 17 years old	0.392(0.821):0.633	−0.739(0.894):0.409	1.010(0.770):0.190	0.094(0.750):0.901	1.126(0.791):0.154
Major illness before 17 years old	1.229(0.736): 0.095	1.955(0.772): 0.011*	2.223(0.885): 0.012*	−0.063(0.713): 0.930	−0.171(0.889): 0.847
Other major upheaval before 17 years old	0.511(0.600): 0.359	−.109(0.532): 0.837	−.327(0.499): 0.512	0.527(0.519): 0.310	0.092(0.611): 0.881
RTES (past 3 yrs)					
Bereavement	−0.358(0.590): 0.544	0.166(0.542): 0.759	−0.485(0.494): 0.326	0.544(0.520): 0.295	−0.862(0.626): 0.169
Major upheaval in relationships	−0.569(1.041): 0.584	1.100(1.025): 0.283	0.468(0.763): 0.539	0.326(0.779): 0.676	1.300(0.791): 0.100
Traumatic sexual abuse	0.903(0.781):0.248	0.313(0.769):0.684	2.007(1.161): 0.084	0.071(0.761): 0.926	−0.622(1.081): 0.565
Nonsexual abuse	0.392(0.892): 0.660	0.514(0.814): 0.528	0.456(0.825): 0.580	0.978(0.826): 0.236	1.311(0.889): 0.140
Extreme illness or injury	1.170(0.725): 0.106	0.985(0.695): 0.168	0.600(0.687): 0.383	−0.610(0.747): 0.414	1.171(1.122): 0.297
Major change in work	0.271(0.592): 0.647	−0.103(0.539): 0.843	0.388(0.496): 0.434	0.638(0.517): 0.217	0.584(0.614): 0.341
Other major upheaval	0.430(0.689): 0.532	−0.224(0.674): 0.740	0.176(0.601): 0.769	−0.002(0.629): 0.998	1.334(1.049): 0.204

*Note*: The Est. in the table stands for the estimated difference in the mean scores of personality dysfunction measures in the logit scale between the presence and absence of the trauma condition. The greater the positive value the greater association between that particular trauma and personality disturbance.

Abbreviation: CTES, Childhood Traumatic Events Scale.

* *p* < 0.02.

** *p* < 0.01.

## DISCUSSION

The strength of this study is in the careful selection of a population with the full range of age and personality pathology rather than the simpler selection of a general group. It nonetheless has limitations as the numbers were small and some important associations may have not been detected. It could also be argued that it is not possible to examine the relationship between trauma and borderline personality disorder in the ICD‐11 classification system as borderline is not represented, except as a pattern specifier (Reed, 2018). But as borderline personality is increasingly noted to be a general rather than a specific diagnosis of PD (Mulder et al., 2016; Mulder & Tyrer, 2023; Sharp & Oldham, 2023), study of its pathology at a domain level is a more pertinent method of investigation. A recent study along these lines examined the effects of trauma on personality found that several domains may mediate the internalizing symptoms that are so prominent in clinical cases of personality disorder (Bach et al., 2022).

The associations between the domains of negative affectivity and detachment and childhood trauma are consistent with the findings of other studies (Berenz et al., 2013; Fossati et al., 1999; Newcomb et al., 2024) but, as with these other findings, the links are relatively modest and not strong enough to indicate an important aetiological link.

These results represent the first study to examine the relationship between the full range of traumatic experiences and PD using the ICD‐11 classification. They confirm that the strongest association is between sexual abuse in childhood and personality problems in adult life, with the negative affective domain having a stronger relationship than other domains, including the combined borderline construct identified in the PAQ‐11. The agreement between recent traumatic events and PD was less significant by comparison, suggesting, if not confirming, that it is early life experiences that are more relevant than later ones in the development of personality disorder. The association between major illness in childhood and subsequent personality disorder with both negative affectivity and Anankastia domains represented is a new finding that needs replication. Major medical illness is clearly one of those significant negative environmental events that can have a long‐term impact on psychopathology (Tyrer et al., 2024).

The important new finding with relevance to ICD‐11 is that sexual abuse specifically associated with the domain of negative affectivity and, by association, with the combined PAQ‐11 features comprising the borderline group. But the findings should be seen in context with the other lesser associations with other domains. It cannot be concluded from the data that there is a specific association between childhood sexual abuse and borderline pathology, and so, the findings do not support the aetiological hypothesis that such abuse is directly linked to this type of personality disturbance. At the same time, the general body of evidence that childhood trauma is more closely linked to personality disorder than other conditions such as depression (Jayakody & Gunadasa, 2023) suggests there is a causative connection in the broadest sense. It is possible that further research into complex post‐traumatic stress disorder may firm up the degree of association, but at present, firm conclusions cannot be made.

## CONCLUSIONS

Childhood trauma overall is related to personality disorder to a greater extent than recent traumatic events. The most prominent association is between sexual abuse in childhood and adult personality disorder with the negative affective domain having stronger associations than other domains.

## CONFLICT OF INTEREST STATEMENT

PT was the Chair of the ICD‐11 Revision Group for the Classification of Personality Disorders and is Chair of the nidotherapy charity, NIDUS‐UK. The other two authors have no interests to declare.

## ETHICS STATEMENT

Ethical approval for the study was granted by The University of Nottingham Research Ethics committee—Ref: FMHS 91‐1022.

## Data Availability

Data and analysis details are available from MY.
